# A sustainable seven-electron cascade battery via orchestrated gas-liquid-solid triphase redox reactions

**DOI:** 10.1126/sciadv.aef2744

**Published:** 2026-07-03

**Authors:** Lingchang Wu, Chaoyi Qiu, Junwei Zhang, Zihao Tao, Xiang Liu, Zhixiao Cai, Haoxiang Yu, Lei Yan, Liyuan Zhang, Ting-Feng Yi, Jie Shu

**Affiliations:** ^1^ School of Chemistry and Chemical Engineering, Shaoxing University, Shaoxing 312000, Zhejiang, China.; ^2^ School of Materials Science and Chemical Engineering, Ningbo University, Ningbo 315211, China.; ^3^ Key Laboratory of Dielectric and Electrolyte Functional Material Hebei Province, School of Resources and Materials, Northeastern University at Qinhuangdao, Qinhuangdao 066004, China.

## Abstract

The quest for high-energy-density batteries has spurred interest in multielectron chemistry beyond conventional two-electron reactions. Here, we report a cascade battery that synergistically integrates gas-phase (Cl_2_ ↔ Cl^−^), liquid-phase (Cu^2+^ ↔ Cu^+^), and solid-phase (S ↔ CuS ↔ Cu_2_S) redox reactions within a deep eutectic solvent (DES) electrolyte. This unique gas-liquid-solid triphase coupling strategy unlocks a seven-electron transfer process. In particular, the chloride-rich DES electrolyte fundamentally alters the copper (Cu) redox thermodynamics, enabling a reversible liquid-phase Cu^2+^/Cu^+^ couple via the formation of stable [CuCl_3_]^2−^ complexes, which prevents disproportionation. The resulting cascade cell delivers an ultrahigh specific capacity of 4426.4 milliampere hours per gram [based on sulfur (S)] and exceptional cycling stability (88.5% capacity retention after 2000 cycles at 10 C). Furthermore, a practical pouch cell configuration achieves a high operating voltage of 1.5 volts and a remarkable energy density of 6917 watt-hours per kilogram (based on S; 2767 watt-hours per kilogram based on the total mass of the cathode), substantially surpassing most aqueous S-based systems. Ultimately, this work underscores that the strategic integration of orchestrated gas-liquid-solid triphase chemistry transcends the capacity limits of conventional single-phase reactions, demonstrating a viable pathway toward a next-generation paradigm for ultrahigh-energy-density storage.

## Teaser

Integrating gas-liquid-solid triphase redox reactions enables ultrahigh-energy-density battery storage.

## INTRODUCTION

The search for next-generation batteries has never stopped. Among the myriad emerging systems, sulfur-based rechargeable batteries are regarded as promising candidates (*1*, *2*). Sulfur serves as a key electrode material that is not only eco-friendly, low-cost, and abundant but also functions as a natural electron acceptor. Through a two-electron transfer process, sulfur achieves a stable octet configuration, enabling a high theoretical capacity of 1675 mA·hour g^−1^ (*3*–*9*). Despite this advantage, the two-electron reaction alone is insufficient for developing high-energy-density sulfur-based batteries.

The construction of multielectron conversion reactions is an effective strategy for enhancing energy density, yet it remains challenging due to the difficulty in activating and stabilizing multiple redox couples within a single system. Recently, an aqueous Cu-S system exhibiting a four-electron redox reaction has been reported, which doubles the theoretical capacity compared to conventional two-electron sulfur redox reactions (S/S^2−^) (*10*–*15*). In this system, Cu^2+^ in the electrolyte shifts from its conventional role as a charge carrier to that of a redox-active participant in electrode reactions, ultimately enabling the transition S ↔ CuS ↔ Cu_2_S through two solid-phase conversions (*16*, *17*). However, this mechanism still exhibits limitations as the redox-active copper species in the electrolyte are not fully used, and activating a direct liquid-phase redox reaction involving the Cu^2+^/Cu^+^ couple could efficiently augment the total capacity.

For this reason, substantial efforts have been made toward stabilizing the Cu^+^ ions in the electrolyte to enable a reversible Cu^2+^/Cu^+^ redox reaction. A prominent strategy involves the use of coordination chemistry, particularly through the introduction of halide ions such as Cl^−^, to modulate the copper ion environment (*18*–*22*). For example, Duan *et al.* demonstrated the effectiveness of this approach in enabling copper conversion chemistry for aqueous batteries, highlighting that the formation of stable copper(I) chloride complexes is crucial for achieving a reversible liquid-phase Cu^2+^/Cu^+^ redox couple (*19*). Zhang *et al.* elucidated the mechanism of the copper(I) chloride system through molecular dynamics (MD) simulations, revealing that specific coordination geometries of chloride ions around Cu^+^ effectively suppress its disproportionation (*21*). Building on this principle, this chloride coordination strategy has been successfully extended to various electrochemical systems, underscoring its broad applicability.

The Cl^−^ ions not only can select as a stabilizer for Cu^+^ but also facilitate an additional energy storage mechanism by enabling a high-potential gas-phase reaction. Typically, Zhang *et al.* successfully used the Cl_2_/Cl^−^ redox couple with high potential to construct a high-voltage aqueous battery (*23*). Similarly, Zhu *et al.* achieved high-capacity Na-Cl_2_ and Li-Cl_2_ batteries by reversibly trapping the Cl_2_ within porous carbon hosts, showcasing a viable strategy to use a gas-phase redox couple (*24*, *25*). Inspired by the previous works, it can be inferred that integrating gas-phase and liquid-phase redox chemistries with the existing solid-phase system offers a promising pathway toward an ideal energy storage system with ultrahigh energy density.

**Fig. 1.**
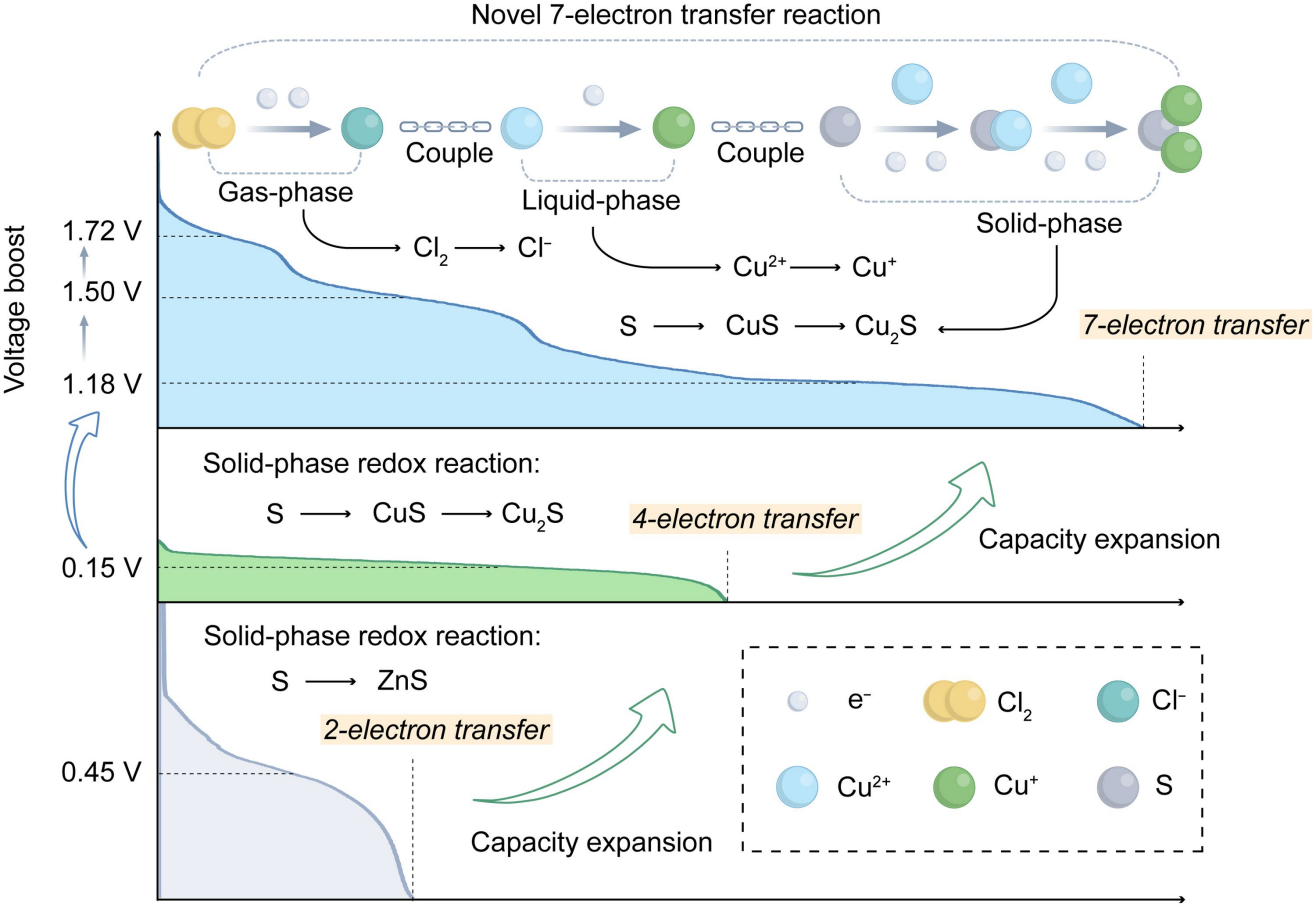
Schematic of the designed cascade battery with gas-phase, liquid-phase, and solid-phase redox reactions based on the seven-electron transfer.

Herein, we design a cascade battery that integrates gas-phase, liquid-phase, and solid-phase conversion reactions, as illustrated in Fig. 1. Within the deep eutectic solvent (DES), composed of choline chloride (ChCl) and urea, a gas-phase reaction (Cl_2_ ↔ Cl^−^), enabled by the high concentration of Cl^−^, first provides a distinct dimension of charge storage. Through a combination of advanced characterization and theoretical calculations, the liquid-phase conversion is verified to realize the Cu^2+^ ↔ Cu^+^ redox pathway. This process leverages the abundant chloride ions to stabilize copper species that are otherwise unstable in conventional aqueous electrolytes. Subsequently, a solid-phase sulfur conversion (S ↔ CuS ↔ Cu_2_S) is seamlessly coupled with the liquid-phase conversion by the remaining Cu^2+^ ions, thereby establishing a gas-liquid-solid triphase cascade system that features a seven-electron transfer, enabled by the maximized utilization of all redox-active species. This holistic integration allows the DES to act as a chemical nexus that orchestrates the interplay between the disparate phases, enabling a continuous electrochemical pathway. In this way, the triphase cascade cell delivers a specific capacity of 4426.4 mA·hour g^−1^ (based on sulfur), surpassing most previously reported sulfur-based cells. It also exhibits an excellent cycling stability, retaining 88.5% capacity retention over 2000 cycles at a rate of 10 C. To evaluate practical applicability, a pouch cell constructed with a sulfur cathode, DES electrolyte, and zinc anode achieves an operating voltage of 1.5 V, substantially higher than existing aqueous sulfur-based systems, along with a high energy density of up to 6917 Wh kg^−1^ (based on sulfur; 2767 Wh kg^−1^ based on the total mass of the cathode).

## RESULTS

### Thermodynamic analysis and electrochemical validation of the Cu^2+^/Cu^+^ redox couple in a DES electrolyte

**Fig. 2.**
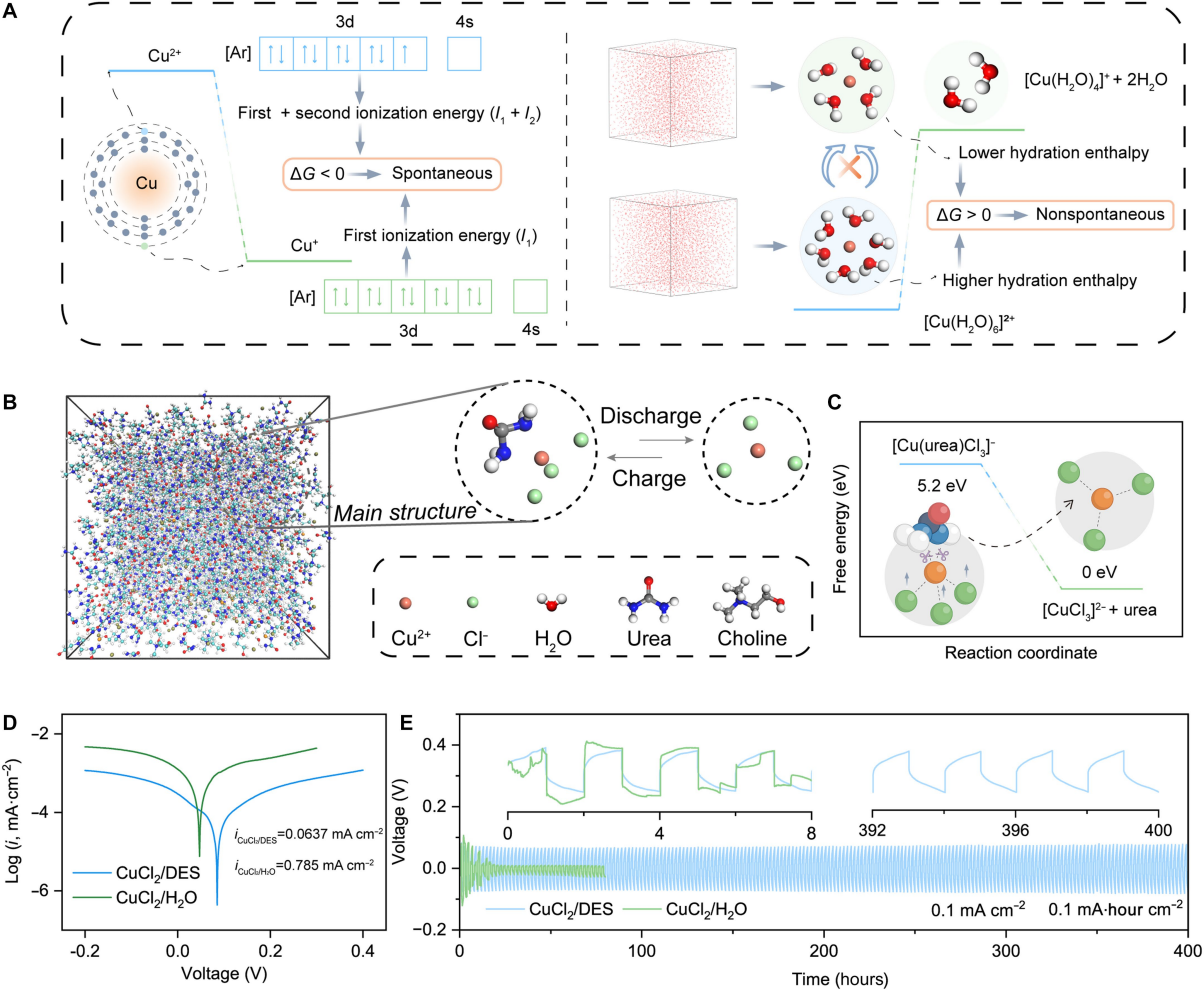
Thermodynamic analysis and electrochemical validation of the Cu^2+^/Cu^+^ redox couple in a DES electrolyte. (**A**) Illustration of the stability of copper ions under vacuum and aqueous solution. (**B**) MD simulations of the representative Cu^2+^ and Cu^+^ solvation structures in the DES. (**C**) Theoretical computations of the reaction pathways for the Cu^2+^ and Cu^+^ in the DES. (**D**) Tafel polarization curves of Cu anodes in CuCl_2_/DES and CuCl_2_/H_2_O. (**E**) Cycling performances of Cu||Cu symmetric cells in CuCl_2_/DES and CuCl_2_/H_2_O at 0.1 mA cm^−2^ with a capacity of 0.1 mA·hour cm^−2^.

To evaluate the feasibility of stabilizing Cu^+^ in the electrolyte, the relative thermodynamic stability of copper ions is first compared by analyzing the interplay between ionization energies and solvation enthalpies. As schematically illustrated in Fig. 2A, in the gas phase, where thermodynamics are dictated solely by electronic structure, the d^10^ configuration of Cu^+^ is substantially more stable than the d^9^ configuration of Cu^2+^, due to the substantially higher second ionization energy of copper relative to the first. However, this thermodynamic preference is reversed in aqueous solution, where the markedly different hydration structures and resulting solvation enthalpies render Cu^+^ unstable and drive its spontaneous disproportionation into Cu^2+^ and Cu^0^. MD simulations (Fig. 2A and fig. S1) confirm that the Cu^2+^, with its d^9^ electronic configuration, adopts a hexacoordinate, distorted octahedral geometry as a direct consequence of the Jahn-Teller effect, which removes electronic degeneracy to stabilize the ion. This tightly bound hydration structure, combined with the high charge density of Cu^2+^, enhances electrostatic interactions with water molecules, leading to a highly exothermic hydration enthalpy. In contrast, the Cu^+^ ion features a spherically symmetric d^10^ configuration, lacks Jahn-Teller distortion, and has lower charge density. Accordingly, it forms a weakly solvated tetracoordinate hydrate (fig. S2), accompanied by a substantially lower hydration enthalpy. Consequently, the large energy gains from hydrating Cu^2+^ more than compensate for the high energy cost of the second ionization. This implies that the thermodynamic landscape can be strategically modulated. For instance, using a DES enables preferential coordination and stabilization of Cu^+^, often through the formation of robust chloro-complexes, thereby altering the thermodynamic landscape to favor the reduction of Cu^2+^.

To gain molecular-level insight into the unique stabilizing effect of DES on Cu^+^, a comparative analysis of the Fourier transform infrared (FTIR) spectra of CuCl_2_/DES and CuCl_2_/H_2_O systems is conducted, revealing critical differences in the local solvation environment of copper ions (fig. S3). Notably, the broad absorption band in the 3650 to 3000 cm^−1^, attributed to O─H and N─H stretching vibrations, is not only centered at a markedly lower wave number in the DES but also exhibits substantially greater intensity (*26*). The substantial red shift and intensity enhancement, relative to the aqueous system, indicate a substantially stronger hydrogen-bonding network within the DES. Such robust intermolecular interactions promote the formation of a highly structured solvation shell, distinct from conventional hydration (*27*).

To further elucidate the underlying stabilization mechanism, MD simulations are used to characterize the primary coordination structures of copper ions, identifying dominant species as [Cu(urea)Cl_3_]^−^ and [CuCl_3_]^2−^ (Fig. 2B and figs. S4 and S5). Subsequent density functional theory (DFT) calculations based on these structures confirm that the reduction process ([Cu(urea)Cl_3_]^−^ + e^−^ → [CuCl_3_]^2−^ + urea) is strongly exergonic, as evidenced by a large negative Gibbs free energy change (Fig. 2C). These results indicate that the unique solvation environment of the DES, enriched with chloride ions, sufficiently stabilizes Cu^+^ to render its formation from Cu^2+^ a spontaneous process.

Building on these insights, the practical performance of the CuCl_2_/DES electrolyte in a symmetrical cell configuration is evaluated, focusing on anode compatibility and long-term cycling stability. Tafel analysis (Fig. 2D) shows that the DES electrolyte effectively suppresses copper corrosion, reducing the corrosion current density to 0.0637 mA cm^−2^, substantially lower than that of the corrosive CuCl_2_/H_2_O electrolyte (0.785 mA cm^−2^). Furthermore, the long-term cycling performance (Fig. 2E) demonstrates exceptional stability for the CuCl_2_/DES system, with the symmetric cell operating steadily for over 400 hours at 0.1 mA cm^−2^ and 0.1 mA·hour cm^−2^, whereas the aqueous system failed during initial cycles. X-ray diffraction (XRD) analysis is performed to elucidate the origin of this performance disparity (fig. S6). The pattern obtained from the copper anode in the failed aqueous cell confirms the presence of CuCl, whereas no such by-products are detected on the anode cycled in the DES electrolyte. This indicates that CuCl formation constitutes the primary failure mechanism in the aqueous system, a detrimental side reaction that is effectively suppressed in the DES. This suppression does not result from simple physical blocking but stems from a fundamentally altered copper redox pathway, specifically the stabilization of Cu^+^ in the liquid phase.

### Working mechanism of the gas-liquid-solid triphase redox cascade cell

With the fundamental viability of the DES electrolyte confirmed for stabilizing copper redox species, the gas-liquid-solid triphase redox cascade cell is constructed by integrating this system with a sulfur cathode. To unravel its complex working mechanism, a series of in situ and ex situ characterizations are systematically conducted.

**Fig. 3.**
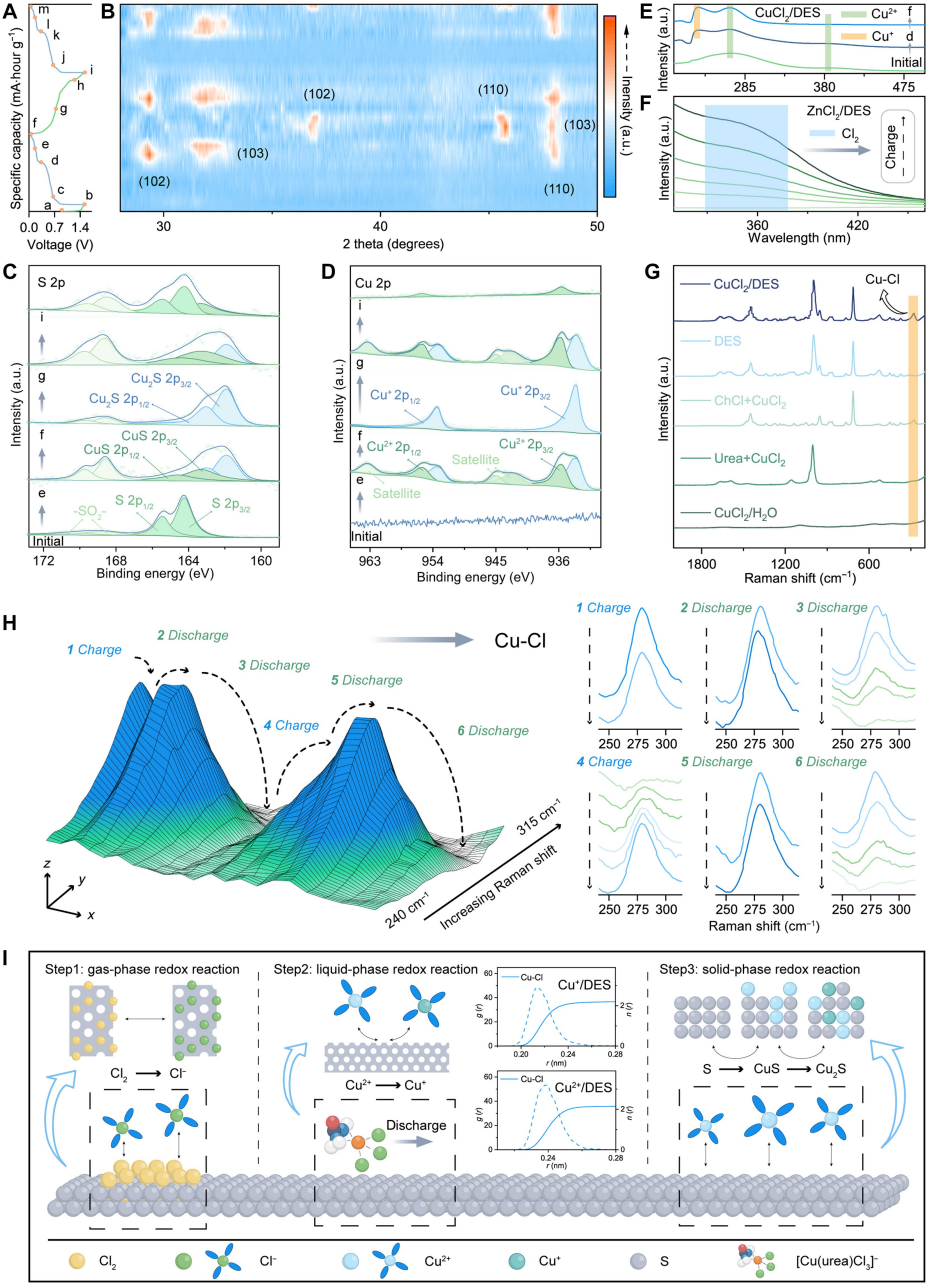
Working mechanism of the gas-liquid-solid triphase redox cascade cell. (**A**) GCD curve of the cascade cell. (**B**) XRD patterns of the S@AC cathode collected in the first two cycles. a.u., arbitrary units. (**C**) S 2p and (**D**) Cu 2p regions of the XPS spectrum for the S@AC cathode at initial (point a), discharged to 0.15 V (point e), fully discharged (point f), charged to 0.7 V (point g), and second cycle fully charged (point i) states. (**E**) UV-Vis spectra of copper species at initial (point a), discharged to 0.45 V (point d), and fully discharged (point f) states in the cascade cell. (**F**) UV-Vis spectra of the ZnCl_2_/DES electrolyte during the charging process. (**G**) Raman spectra of a series of electrolytes. (**H**) Raman spectra of the electrolyte collected in the first two cycles. (**I**) Schematic of the gas-phase, liquid-phase, and solid-phase redox reaction process. The inset in (I) shows the RDF of the Cu^+^ and Cu^2+^ in the DES.

Initially, XRD and x-ray photoelectron spectroscopy (XPS) are conducted to gain insight into the mechanism of the cascade cell. During the galvanostatic charge-discharge (GCD) cycling at 0.2 C (Fig. 3A), XRD patterns collected at various voltages (Fig. 3B) reveal that, during the initial charging stage and the subsequent discharge down to 0.45 V (point d), no new solid-phase diffraction peaks emerge, suggesting that the reaction proceeds primarily in the gas or liquid phase. Upon further discharging, distinct peaks appear at 29.2°, 31.7°, and 47.9°, which can be indexed to the (102), (103), and (110) planes of CuS (JCPDS no. 78-0876; fig. S7). As the discharge continues, these CuS peaks gradually diminish, whereas new reflections emerge at around 37.2°, 45.7°, and 48.1°, corresponding to the (102), (110), and (103) planes of Cu_2_S (JCPDS no. 84-0207; fig. S8). This phase evolution is reversible upon charging: The Cu_2_S peaks disappear, CuS reemerges, and last, all solid-phase signals vanish by 1.3 V (point h), indicating a return to non-solid-phase processes. The second discharge profile closely mirrors the first, confirming high reversibility.

To further elucidate the solid-phase transitions inferred from XRD, XPS is performed at key voltages (Fig. 3, C and D). Upon discharging to 0.15 V (point e), the S 2p spectrum exhibits new peaks that are distinct from those of pristine elemental sulfur (165.48 and 164.28 eV). Specifically, the peaks at 164.58 and 163.28 eV correspond to S^2−^ in CuS, whereas those at 162.98 and 161.88 eV are assigned to S^2−^ in Cu_2_S. Correspondingly, the Cu 2p spectrum reveals the presence of both Cu^2+^ (peaks at 963.48, 955.58, 945.08, 942.68, and 935.68 eV) and Cu^+^ (peaks at 953.48 and 933.68 eV) (*12*, *15*). At the fully discharged state (point f), the signals for CuS vanish, confirming that Cu_2_S is the final discharge product and CuS is an intermediate in the solid-phase conversion. During the subsequent charging process, this transformation reverses, with the characteristic signals for CuS and elemental sulfur reappearing sequentially. Upon full charge (point i), the spectra are dominated by signals from elemental sulfur, with only a trace amount of CuS detected, suggesting a high degree of reversibility. Collectively, these XRD and XPS results corroborate a reversible, four-electron phase conversion pathway (S ↔ CuS ↔ Cu_2_S) driven by the Cu^2+^/Cu^+^ and S/S^2−^ redox couples.

Meanwhile, ultraviolet-visible (UV-Vis) spectroscopy is used to monitor the evolution of redox-active species at various voltages (Fig. 3, E and F). The initial spectrum displays two characteristic absorption peaks near 269 and 398 nm, attributable to Cu^2+^ species, which persist throughout the electrochemical process, confirming the continuous presence of Cu^2+^ in the electrolyte (fig. S9) (*28*). Upon discharging into the second stage, a new absorption feature emerges at around 228 nm and remains until the end of discharge. This peak aligns with that of the CuCl/DES reference electrolyte (fig. S10), identifying it as Cu^+^. Although the evolution of copper species is central to the redox mechanism, the origin of the first high-potential discharge plateau remains unexplained by copper redox alone, strongly implying the involvement of an additional redox couple. To identify this process, a control experiment is performed using a copper-free ZnCl_2_/DES electrolyte. During charging, a distinct absorption peak attributed to Cl_2_ appears in the range of 320 to 420 nm and intensifies with further charging, thus confirming its role as the active species responsible for this plateau. Furthermore, the reversibility of the process is demonstrated by monitoring the UV-Vis signals during the subsequent discharge stage (fig. S11). As discharging proceeds, the characteristic absorption of Cl_2_ systematically decreases, providing direct spectroscopic evidence that the Cl_2_ is effectively reduced back to Cl^−^.

To further investigate the practical implications of the gas-phase stage, the gas production is first quantified using differential electrochemical mass spectrometry (DEMS). The results show that the total amount of gas detected during the charging process is only 6.8 μmol (fig. S12), confirming the successful conversion of Cl^−^ to Cl_2_ at the cathode. Despite the generation of Cl_2_, in situ pressure measurements demonstrate that, in the absence of external gas flow interference, the internal cell pressure remains remarkably stable, with an increase of only 1.1 kPa throughout the gas production process (fig. S13). This negligible pressure variation is directly attributed to the superior storage capability of the microporous carbon. As evidenced by Brunauer-Emmett-Teller (BET characterization (fig. S14), the host has an ultrahigh specific surface area (2860.6 m^2^ g^−1^) and a large total pore volume (1.44 cm^3^ g^−1^). The typical type I(b) isotherm profile confirms a predominant microporous structure, which provides a strong confinement effect that allows the formed Cl_2_ to be effectively captured and stored via physical and chemical adsorption rather than existing as free gas. This structural design effectively ensures the stability of the gas-phase reaction and lays the foundation for its high reversibility.

To further probe the evolving coordination environment of the electrolyte, Raman spectroscopy is conducted. The initial spectrum exhibits a prominent peak within the range of 250 to 325 cm^−1^, attributed to a Cu-Cl coordination complex formed between Cu^2+^ and ChCl (Fig. 3G) (*23*). As shown in Fig. 3H, throughout the initial charging process, the intensity of the peak decreases, reflecting the consumption of Cl^−^ during Cl_2_ evolution. During subsequent discharge, the intensity recovers as Cl_2_ is reduced back to Cl^−^, replenishing the Cl^−^ available for coordination. Further discharge to the completion of the liquid-phase reaction induces minimal change in intensity, consistent with the similar coordination numbers (CNs) of Cu^+^ and Cu^2+^ chloro-complexes, a finding corroborated by radial distribution function (RDF) analysis (inset of Fig. 3I). Last, during the full discharge process, the onset of solid-phase conversion consumes substantial Cu^2+^ from the electrolyte, resulting in a sharp decline in Raman peak intensity. Throughout subsequent cycles, the Raman intensity profile remains highly reproducible, underscoring the reversible consumption and regeneration of both copper and chlorine species and affirming the exceptional reversibility of the overall process.

Therefore, as depicted in Fig. 3I, the mechanism comprises three sequential stages: an initial gas-phase reaction, a subsequent liquid-phase reaction, and a final solid-phase reaction. The corresponding electrochemical reactions are presented as follows

$$\text{Step}\;1,\;\text{gas-phase:}\;{\text{Cl}}_{2}↔{\text{Cl}}^{-}$$

$$\text{Step}\;2,\;\text{liquid-phase:}\;{\text{Cu}}^{2+}↔{\text{Cu}}^{+}$$

$$\text{Step}\;3,\;\text{solid-phase:}\;S↔\text{CuS}↔{\text{Cu}}_{2}S$$

These integrated processes collectively drive the seven-electron transfer within the Cu-S cascade cell, which can be represented by the following reversible equations

$$\text{Cathode:}\;{\text{Cl}}_{2}+{\text{3Cu}}^{2+}+S+{\text{7e}}^{-}↔{\text{2Cl}}^{-}+{\text{Cu}}^{+}+{\text{Cu}}_{2}S$$

$$\text{Anode:}\;\text{Cu}↔{\text{Cu}}^{2+}+{\text{2e}}^{-}$$

$$\text{Overall:}\;{\text{2Cl}}_{2}+\text{7Cu}+\text{2S}↔{\text{4Cl}}^{-}+{\text{2Cu}}^{+}+{\text{2Cu}}_{2}S+{\text{Cu}}^{2+}$$

### The electrochemical behaviors of the cascade cell

In situ electrochemical impedance spectroscopy (EIS) is used to probe the interfacial dynamics associated with gas-phase, liquid-phase, and solid-phase reactions in the Cu-C cell and the cascade cell (figs. S15 and S16). During charging, the impedance of the cascade cell initially increases, then decreases, before rising sharply at the end. This final rise, common to both cells, is attributed to gas-phase formation. In contrast, the intermediate decrease is absent in the Cu-C cell, confirming that this impedance fluctuation is a characteristic signature of the solid-phase reaction. Moreover, the Cu-C cell exhibits consistently lower impedance throughout the process, which can be explained by the inherently lower resistance of the liquid-phase reaction and the modified electrochemical behavior resulting from the sulfur-coated electrode interface in the cascade cell.

**Fig. 4.**
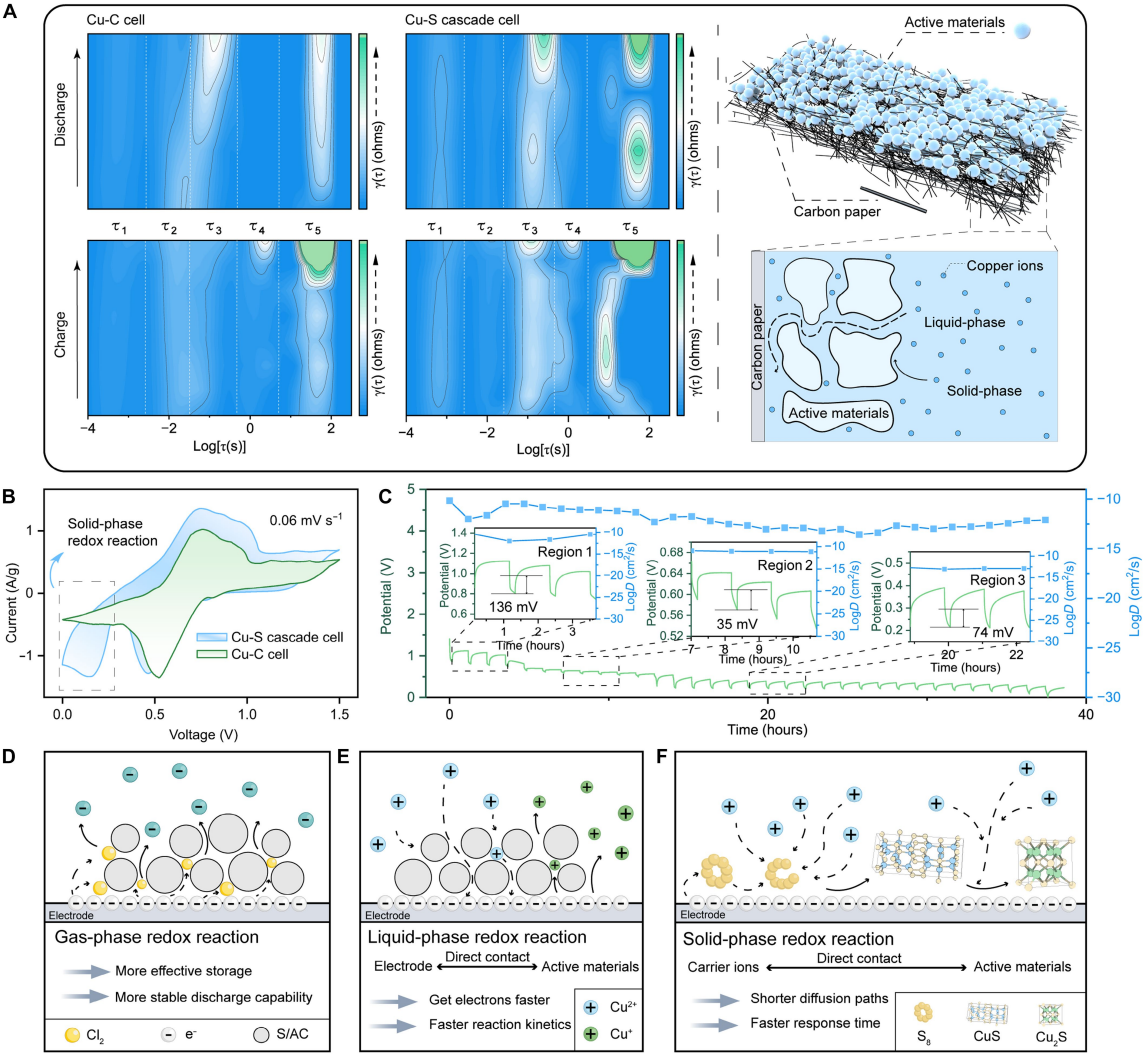
Electrochemical behaviors of the cascade cell. (**A**) DRT at different voltages in the Cu-C cell and cascade cell. The inset in (A) is a schematic of the cathode surface and ion migration within the cathode. (**B**) CV curves of the cascade cell and Cu-C cell. (**C**) Diffusion coefficients and voltage profiles of the cascade cell during GITT measurement. (**D**) Schematic of the gas-phase redox reaction process and its kinetic advantages. (**E**) Schematic of the liquid-phase redox reaction process and its kinetic advantages. (**F**) Schematic of the solid-phase redox reaction process and its kinetic advantages.

To further elucidate interfacial changes, distribution of relaxation time (DRT) analysis is conducted (Fig. 4A). This technique deconvolutes different electrochemical processes by their characteristic time constants (τ), which reflect intrinsic properties such as grain boundary effects, bulk ion conduction, and interfacial phenomena over a broad frequency range, and are closely linked to cell performance. On the basis of the DRT results, the electrochemical processes can be segmented into five distinct regions: τ_1_ (<10^–2.5^ s), τ_2_ (~10^–2.5^ to 10^–1.5^ s), τ_3_ (~10^–1.5^ to 10^-0.3^ s), τ_4_ (~10^–0.3^ to 10^0.5^ s), and τ_5_ (>10^0.5^ s). These regions are respectively attributed to ionic transport within the SEI layer (τ_1_), mass transfer in electrode pores (τ_2_ and τ_3_), the charge-transfer reaction (τ_4_), and diffusion impedance (τ_5_) (*29*, *30*).

During the charge and discharge process, the peaks associated with τ_1_ and τ_2_ exhibit high stability and similar positioning in both cells, indicating comparable behaviors in SEI properties and anode side mass transport. The τ_3_ peak reflects ion transport at the cathode interface. In the Cu-C cell, τ_3_ merges with τ_2_ at higher frequencies, suggesting rapid and direct ion access at the electrolyte-cathode interface. In contrast, the cascade cell shows a τ_3_ peak at lower frequencies, indicating a more complex ion pathway (inset of Fig. 4A), likely due to ion diffusion through the porous solid sulfur coating, whose morphology is corroborated by confocal laser scanning microscopy (CLSM) (fig. S17). In addition, the reaction sequence of the cascade cell during charging is clearly elucidated by the dynamics of the τ_4_ and τ_5_ peaks. The process commences with the liquid-phase reaction, which then persists alongside the subsequently initiated solid-phase reaction, and concludes with the gas-phase reaction at higher potentials. Evidently, for these peaks, the comparisons between the two cells reveal that the changes in intensity and shifts in frequency observed in the cascade cell are direct signatures of the multiphase reaction occurring.

To further observe the distinct electrochemical features during discharge, cyclic voltammetry (CV) is performed on both the Cu-C cell and the cascade cell (Fig. 4B). In the cathodic scan between 0.26 and 1.5 V, both systems exhibit similar electrochemical behavior, consistent with shared gas-phase and liquid-phase reactions. In contrast, the cascade cell shows a pronounced cathodic peak below 0.26 V, which is entirely absent in the Cu-C cell, confirming the successful integration of a solid-phase conversion pathway unique to the cascade system.

Galvanostatic intermittent titration technique (GITT) is used to further analyze the cathode voltage response, revealing three distinct regions (Fig. 4C). Region 1, corresponding to the gas-phase reaction, demonstrates the highest *IR* drop of 136 mV. Region 2, associated with the liquid-phase reaction, shows a moderate *IR* drop of 35 mV, whereas region 3, representing the solid-phase reaction, displays a substantial *IR* drop of 74 mV. The substantially higher *IR* drops for the gas-phase and solid-phase reactions, compared to the liquid-phase process, reflect the greater interfacial resistance caused by gas consumption within a porous framework and the nucleation of a new solid phase, corroborating the proposed mechanism (*25*, *31*).

Further investigation at higher current densities highlights the distinct kinetic characteristics of the three reaction phases (figs. S18 to S20). Although the liquid-phase and solid-phase reactions exhibit a slight, anticipated increase in overpotential in alignment with standard Butler-Volmer kinetics, the gas-phase consumption reaction displays a modest decrease. Such a distinctive trend is attributable to the increased electrochemical driving force at higher rates, which facilitates the desorption of Cl_2_ from the porous carbon host. Collectively, these results underscore the reasonable kinetics of the entire cascade system.

In summary, comprehensive electrochemical analysis confirms that the cascade cell operates via a mechanism that synergistically combines gas-phase, liquid-phase, and solid-phase reactions, as illustrated schematically in Fig. 4 (D to F). The gas-phase reaction enables stable discharge performance, benefiting from effective confinement of active species within the carbon host. The liquid-phase reaction demonstrates accelerated kinetics due to rapid electron transfer, facilitated by direct electronic contact between active materials and the electrode through the porous sulfur coating, quantitatively evidenced by its low impedance and minimal *IR* drop. The solid-phase reaction contributes a rapid response, supported by readily accessible reaction sites that enable short, efficient ion diffusion paths, as indicated by DRT analysis.

### Electrochemical performance of the cascade cell

**Fig. 5.**
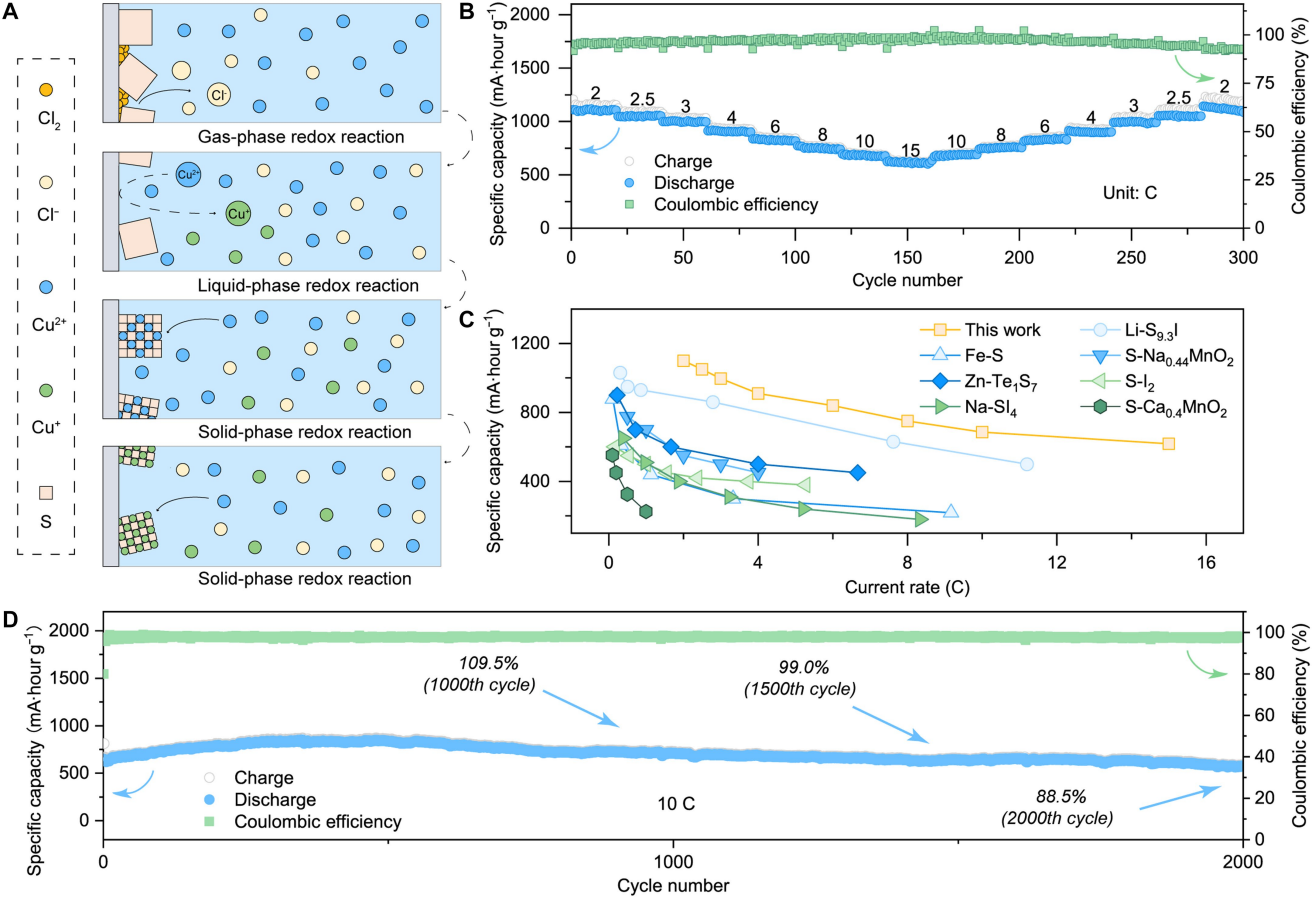
Electrochemical performance of the cascade cell. (**A**) Schematic of the coupling mechanism among the gas-phase, liquid-phase, and solid-phase redox reactions within the cascade cell. (**B**) Rate performance of the cascade cell. (**C**) Comparison of the rate performance for different sulfur-based cells. (**D**) Long-term cycling performance of the cascade cell at 10 C.

Figure 5A schematically illustrates the coupling mechanism integrating gas-phase, liquid-phase, and solid-phase conversions in the cascade cell. On the basis of this well-defined reaction pathway, the electrochemical performance of the cell is systematically evaluated. As depicted in Fig. 5B and fig. S21, the cascade cell demonstrates remarkable rate capability from 2 to 15 C. Specifically, the GCD curves at various current rates exhibit distinct voltage plateaus corresponding to the gas-phase, liquid-phase, and solid-phase redox processes. Notably, the Coulombic efficiency of the gas-phase component demonstrates a generally upward trend as the current rate increases. This phenomenon is primarily attributed to the facilitated gas desorption at higher current densities. Furthermore, it delivers a specific capacity of 625.25 mA·hour g^−1^ (based on sulfur) even at a rate of 15 C. Upon returning to 2 C, the capacity recovers to 97.9% of its initial capacity, indicating excellent electrochemical reversibility. A comparative rate performance analysis with other representative sulfur-based systems, including Fe-S, Zn-S, Na-S, Li-S, Ca-S, and S-I_2_ systems, further highlights the superiority of the cascade cell (Fig. 5C) (*5*, *32*–*37*). Among these systems, the cascade cell shows substantially enhanced rate stability, underscoring its potential as a high-performance energy storage device.

Moreover, the cascade cell also displays excellent cycling stability, as shown in fig. S22. At a rate of 1 C, it sustains a specific capacity of 1744.3 mA·hour g^−1^ after 150 cycles, corresponding to an exceptional retention of 96.0%. Even when cycled at 10 C, the cascade cell maintains a reversible capacity of 574.3 mA·hour g^−1^ after 2000 cycles, achieving a capacity retention of 88.5% (Fig. 5D). Cumulatively, these results confirm the outstanding rate capability and cycling performance of the cascade cell.

### Practical application of the cascade battery

**Fig. 6.**
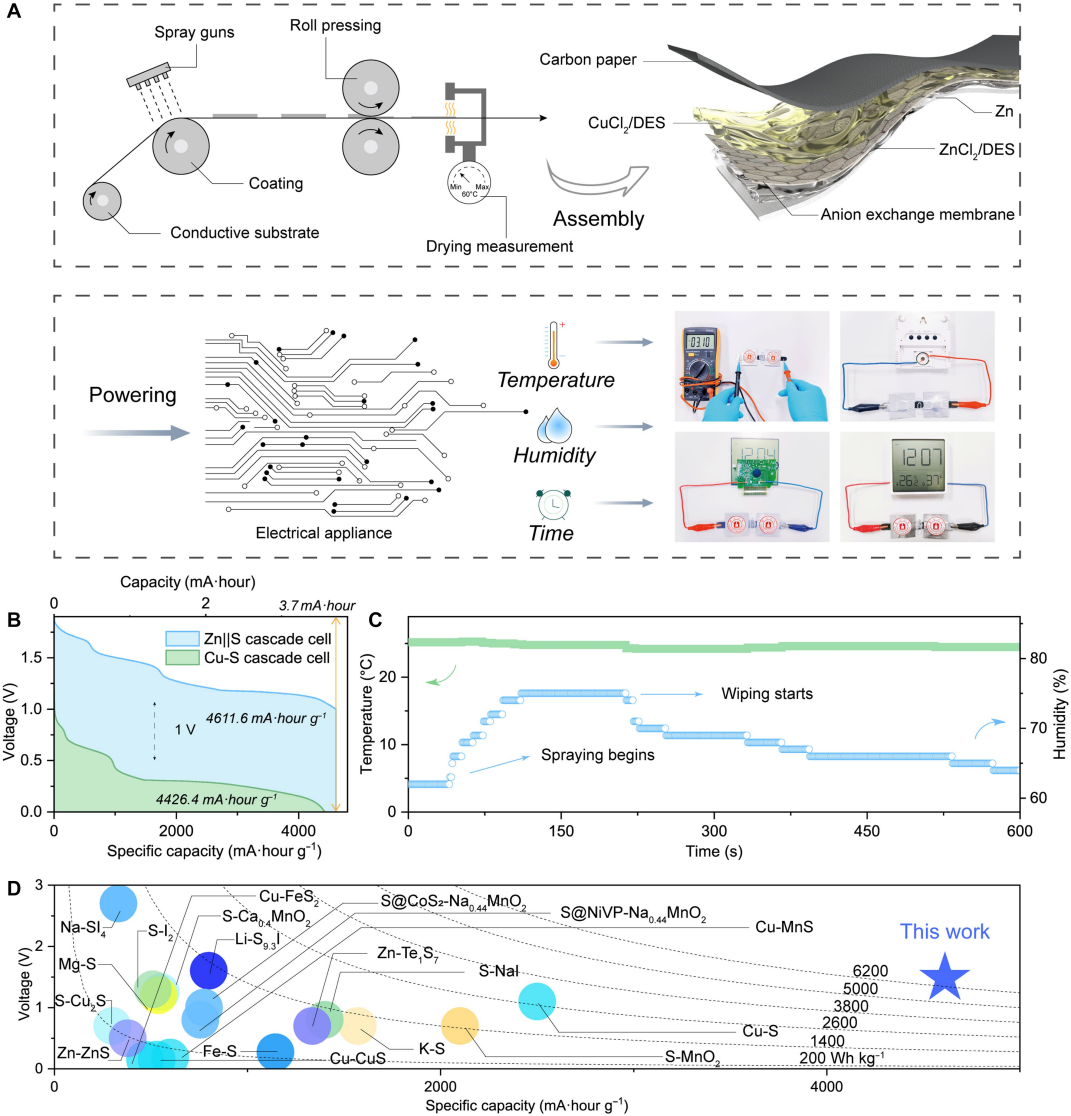
Electrochemical application of the cascade battery. (**A**) Schematic of the continuous fabrication and application of the Zn||S cascade cell. (**B**) GCD curve of the Cu-S cascade cell and Zn||S cascade cell. (**C**) Temperature and humidity changes during 10 min of battery powering the sensor. (**D**) Energy density comparison of the Zn||S cascade cell with other sulfur-based batteries.

To further assess practical application, a full cell (S|CuCl_2_||ZnCl_2_|Zn) incorporating decoupling technology is fabricated as a pouch cell. Figure 6A provides a schematic illustration of the entire workflow, from the fabrication of the electrodes through essential processes such as coating, roll pressing, and drying, to the final application of the assembled pouch cell. The resulting pouch cell exhibits a stable open-circuit voltage (OCV) of ~1.55 V. Furthermore, a battery pack consisting of three such cells connected in series delivers an OCV of 4.6 V (fig. S23), which confirms the reliable scalability from individual units to integrated packs. Beyond the initial OCV, the cell maintains a robust average operating voltage of about 1.50 V during discharge, which is nearly 1 V higher than the voltage plateau observed in the corresponding Cu-S cascade cell (Fig. 6B). This voltage advancement is part of a systematic evolution from the foundational Cu-C and Cu-S systems to the final high-performance Zn||S configuration, whose comparative voltage-time profiles and corresponding cell configurations are collectively summarized in fig. S24 and table S1. Essentially, the substantial voltage enhancement is primarily realized by pairing the low-potential Zn anode with the cascade cathode within the decoupled architecture. Specifically, the underlying electrochemical processes are governed by the following reactions

$$\text{Cathode:}\;{\text{Cl}}_{2}+{\text{3Cu}}^{2+}+S+{\text{7e}}^{-}↔{\text{2Cl}}^{-}+{\text{Cu}}^{+}+{\text{Cu}}_{2}S$$

$$\text{Anode:}\;\text{Zn}↔{\text{Zn}}^{2+}+{\text{2e}}^{-}$$

$$\text{Overall:}\;{\text{2Cl}}_{2}+\text{7Zn}+{\text{6Cu}}^{2+}+\text{2S}↔{\text{7Zn}}^{2+}+{\text{4Cl}}^{-}+{\text{2Cu}}^{+}+{\text{2Cu}}_{2}S$$

In addition to the high operating voltage, the pouch cell demonstrates robust electrochemical stability. As depicted in fig. S25, the cell maintains stable capacity delivery across current rates from 0.8 to 2.5 C, with a high-capacity recovery of 97.1% upon returning to 0.8 C. Furthermore, the pouch cell exhibits excellent long-term durability, retaining 81.5% of its initial capacity after 300 cycles (fig. S26).

Supported by this high-voltage capability and remarkable stability, the cell can power various electronic devices. For instance, an individual pouch cell is sufficient to operate a digital timer (fig. S27). Furthermore, by connecting two cells in series, the cascade battery provides continuous and stable power required for a temperature and humidity sensor, as confirmed by the long-term test presented in Fig. 6C. In addition, a comparative analysis with previously reported sulfur-based batteries highlights the superior performance of this system (Fig. 6D) (*5*, *15*, *32*–*48*). Among these systems, this work exhibits the highest specific capacity and operating voltage, achieving an experimental energy density of 6917 Wh kg^−1^ (based on sulfur; 2767 Wh kg^−1^ based on the total mass of the cathode). The experimental value represents a high utilization of ~78.8% relative to the theoretical energy density (8778 Wh kg^−1^, based on sulfur), which reveals the potential as a high-performance energy storage system.

## DISCUSSION

In summary, we successfully engineer a gas-liquid-solid triphasic cascade system that unlocks a seven-electron transfer. The breakthrough is enabled by the mechanism that substantially enhances the overall utilization of active materials by sequentially coupling the unique gas-phase (Cl_2_ ↔ Cl^−^), liquid-phase redox pathway (Cu^2+^ ↔ Cu^+^), and conventional solid-phase sulfur conversion (S ↔ CuS ↔ Cu_2_S), a process critically enabled by the dual role of Cl^−^ as both the gas-phase active species and a liquid-phase stabilizer of Cu^+^. As a result, the cascade cell displays an outstanding specific capacity of 4426.4 mA·hour g^−1^ and excellent electrochemical stability, with good capacity retention of 88.5% after 2000 cycles at a rate of 10 C. Moreover, the pouch cell based on the sulfur cathode, DES electrolyte, and Zn anode achieves an operating voltage of 1.5 V and a remarkable energy density of 6917 Wh kg^−1^ (based on sulfur; 2767 Wh kg^−1^ based on the total mass of the cathode). Consequently, the cascade battery presents a promising pathway for the development of next-generation high-performance aqueous batteries.

## MATERIALS AND METHODS

### Materials

All chemicals, including S (Sinoreagent), CuCl_2_·2H_2_O (Sinoreagent), CuCl (Aladdin), CuSO_4_ (Sinoreagent), ZnCl_2_ (Aladdin), urea (Aladdin), ChCl (Aladdin), and activated carbon (AC, Aladdin), were used without further purification.

### Synthesis of S@AC

The S@AC was prepared by the melt-diffusion method. Typically, 1.0 g of S was first ground with 1.0 g of AC in a mortar for 30 min, and then the mixture was ball milled for 5 hours at a rotating speed of 300 rpm. The composite was sealed in an autoclave and held at 155°C for 12 hours to yield S@AC.

### Synthesis of DES electrolytes

The DES was prepared by mixing ChCl with urea at a molar ratio of 1:2 and stirring at 60°C for 12 hours. Then, the CuCl_2_/DES and ZnCl_2_/DES solutions were prepared by adding 0.5 M CuCl_2_·2H_2_O and ZnCl_2_ into the obtained DES.

### Material characterization

The morphologies of the electrode surface were confirmed by CLSM (LEXT OLS5100). The structure of products was measured by XRD (Bruker, D8 Advance) and XPS (Thermo Fisher Scientific K-Alpha+). Raman (Thermo Fisher Scientific DXR3) and UV-Vis spectrometer (TU-1950) were used to characterize the chemical information of the electrolyte. DEMS (QAS100-Li) was used to trace gas evolution during battery operation.

### Electrochemical measurements

The working electrode was composed of S@AC, acetylene black, and polytetrafluoroethylene (PTFE) (8:1:1 by weight). The active sulfur mass loading was maintained at 0.8 to 1.6 mg cm^−2^, corresponding to a total cathode loading of 2 to 4 mg cm^−2^. The cascade cell was assembled by using S@AC as the cathode, Cu foil as the anode, 0.5 M CuCl_2_/DES as the electrolyte, and Whatman glass fiber as the separator. The pouch cell was used using Zn metal as the anode with ZnCl_2_/DES as the electrolyte on the anode side and CuCl_2_/DES as the electrolyte on the cathode side, delivering a total capacity of ~3.7 mA·hour. In the GITT test, the cell was performed with the voltage range of 0 to 1.5 V versus Cu^2+^/Cu. The duration time for each applied galvanostatic current was 10 min, followed by an hour of relaxation. GCD cycling was performed using a LANHE battery tester (CT2001A). CV was performed by a BioLogic VSP electrochemical workstation. EIS and Tafel analysis were performed by a CHI760E electrochemical workstation.

### MD simulations

MD simulations were conducted using the GROMACS 2020.6 software package (*49*). The optimized potentials for liquid simulations-all atom (OPLS-AA) force field was used for all components in the system (*50*). Parameters for urea and ChCl were generated via the LigParGen web server (*51*–*53*). The nonbonded parameters for the copper ions were adopted from the particle mesh Ewald (PME)–compatible force field sets developed by Li *et al.* and Ponomarev *et al.* (*54*, *55*). Standard OPLS-AA parameters, as implemented in GROMACS, were used for chloride ions and water, with the latter represented by the TIP3P model (*56*).

The initial simulation systems were constructed using the Packmol software package (*57*). The energy minimization used the steepest descent algorithm to remove unfavorable steric clashes. Subsequently, the system was equilibrated sequentially in the canonical (NVT) and isothermal-isobaric (NPT) ensembles.

Throughout the simulations, the temperature was maintained using the V-rescale thermostat, and the pressure was controlled with the Parrinello-Rahman barostat (*58*, *59*). Periodic boundary conditions were applied in all three dimensions. Long-range electrostatic interactions were treated using the PME method (*60*). A short-range cutoff was used for the van der Waals interactions, managed with a Verlet buffer scheme, and long-range dispersion corrections were applied for both energy and pressure (*61*). All bonds involving hydrogen atoms were constrained with the LINCS algorithm (*62*).

Following equilibration, a production MD run was performed in the NPT ensemble, from which system coordinates were saved periodically for subsequent analysis. The local solvation structure around the copper ion was characterized by calculating the RDF, from which the first-shell CNs were determined.

### DFT calculations

The Gaussian 16 software package was used to perform all DFT calculations. The B3LYP hybrid functional (*63*, *64*) was used in conjunction with the def2-TZVP basis set for all atoms (*65*).
