# Supplementary material for: A sustainable seven-electron cascade battery via orchestrated gas-liquid-solid triphase redox reactions
Source: Sci Adv. 2026 Jul 3;12(27):eaef2744. doi: 10.1126/sciadv.aef2744 (PMC13330902; doi:10.1126/sciadv.aef2744)
Supplement: Supplementary file 1 — Supplementary Text Figs. S1 to S27 Table S1 [file sciadv.aef2744_sm.pdf]

Science Advances
AAAS

Supplementary Materials for

A sustainable seven-electron cascade battery via orchestrated gas-liquid-solid triphase redox reactions

Lingchang Wu *et al.*

Corresponding author: Haoxiang Yu, yuhaoxiang@nbu.edu.cn; Ting-Feng Yi, tfyihit@163.com; Jie Shu, shujie@nbu.edu.cn

Sci. Adv. **12**, eaef2744 (2026)
DOI: 10.1126/sciadv.aef2744

This PDF file includes:

Supplementary Text
Figs. S1 to S27
Table S1

Supplementary Text

Calculation of Theoretical Specific Capacity

1. Theoretical Specific Capacity of the Electrode:

$$C_{th,\ electrode} = (n \times F) / (3.6 \times M)$$

Where:
n = 7 (total electrons transferred per S atom in the cascade reaction)
F = 96485 C mol^{-1} (Faraday constant)
M(S) = 32.06 g mol^{-1} (Molecular weight of Sulfur)
The resulting $C_{th,\ electrode}$ = 5851.8 mAh g^{-1} (based on sulfur)

2. Theoretical Specific Capacity of the Cell:

$$C_{th,\ cell} = (n \times F) / (3.6 \times M)$$

Where:
n = 7
F = 96485 C mol^{-1}
M(S) = 32.06 g mol^{-1}
M(Zn) = 65.38 g mol^{-1} (Molecular weight of zinc)
M($CuCl_2$) = 134.45 g mol^{-1} (Molecular weight of copper(II) chloride)
M = M(S) + 3.5M(Zn) + 3M($CuCl_2$) = 664.24 g mol^{-1} (Total molecular weight)
The resulting $C_{th,\ cell}$ = 282.4 mAh g^{-1}

Calculation of Theoretical Energy Density

A strictly thermodynamic calculation of the theoretical cell voltage based on standard reduction potentials is challenging due to the non-ideal nature of the DES. Therefore, to provide a meaningful and practical benchmark, the theoretical energy density was evaluated using the experimental average operating voltage (V_{avg} = 1.5 V):

Experimental average voltage: V_{avg} = 1.5 V

Theoretical specific capacity: C_{th} = 5851.8 mAh g^{-1} (based on sulfur)

Theoretical Energy Density: $E_{th} = C_{th} \times V_{avg}$ = 5851.8 × 1.5 = 8778 Wh kg^{-1}

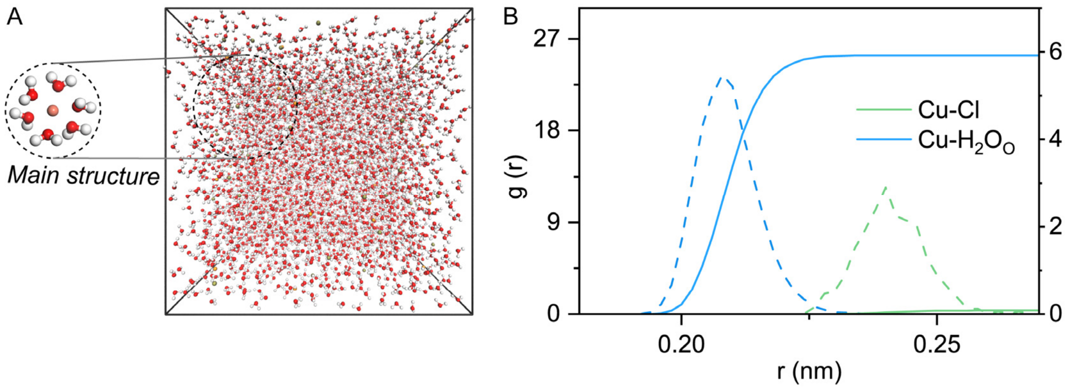

Fig. S1. Thermodynamic analysis and electrochemical validation of the Cu^{2+}/Cu^{+} redox couple in a DES electrolyte. (**A**) The solvation structures of the $CuCl_2/H_2O$. Atom color: Cu, orange; Cl, tan; H, white; O, red. (**B**) The resulting RDF g(r) and coordination number N(r) in the $CuCl_2/H_2O$.

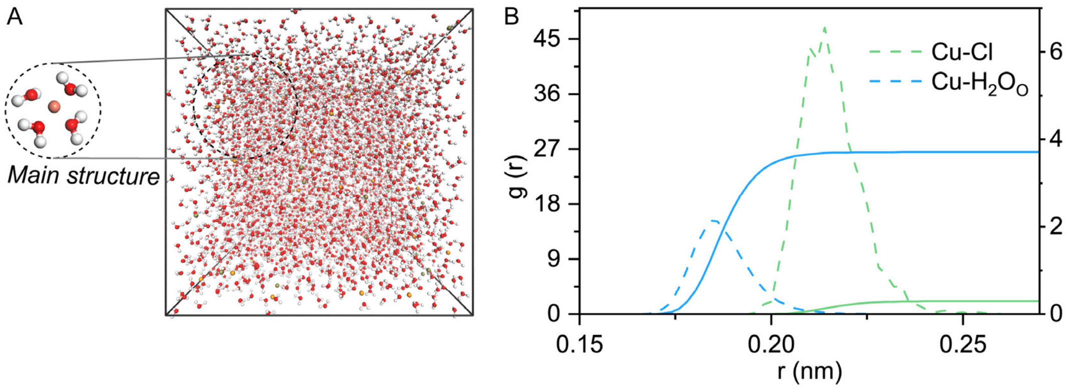

Fig. S2. Thermodynamic analysis and electrochemical validation of the Cu^{2+}/Cu^{+} redox couple in a DES electrolyte. (**A**) The solvation structures of the CuCl/H_2O. Atom color: Cu, orange; Cl, tan; H, white; O, red. (**B**) The resulting RDF g(r) and coordination number N(r) in the CuCl/H_2O.

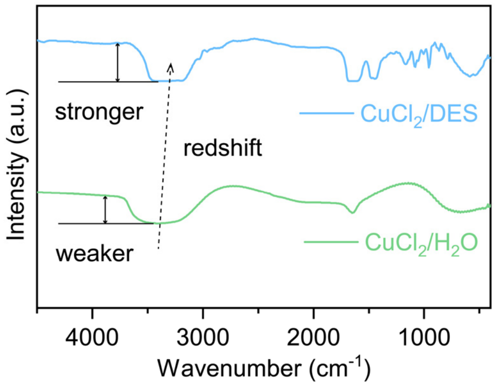

Fig. S3. Thermodynamic analysis and electrochemical validation of the Cu^{2+}/Cu^{+} redox couple in a DES electrolyte. Fourier-transform infrared spectroscopy of the $CuCl_2$/DES and $CuCl_2$/H_2O.

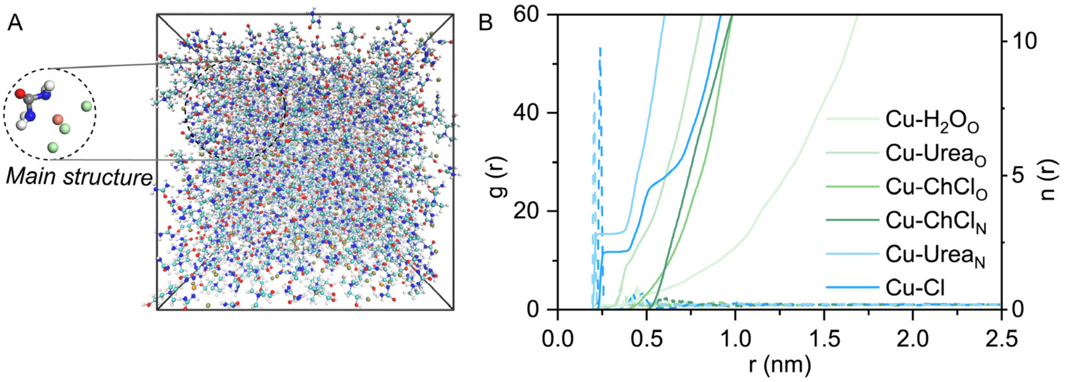

Fig. S4. Thermodynamic analysis and electrochemical validation of the Cu^{2+}/Cu^{+} redox couple in a DES electrolyte. (**A**) The solvation structures of the $CuCl_2$/DES. Atom color: Cu, orange; Cl, tan; N, blue; C, gray; H, white; O, red. (**B**) The resulting RDF g(r) and coordination number N(r) in the $CuCl_2$/DES.

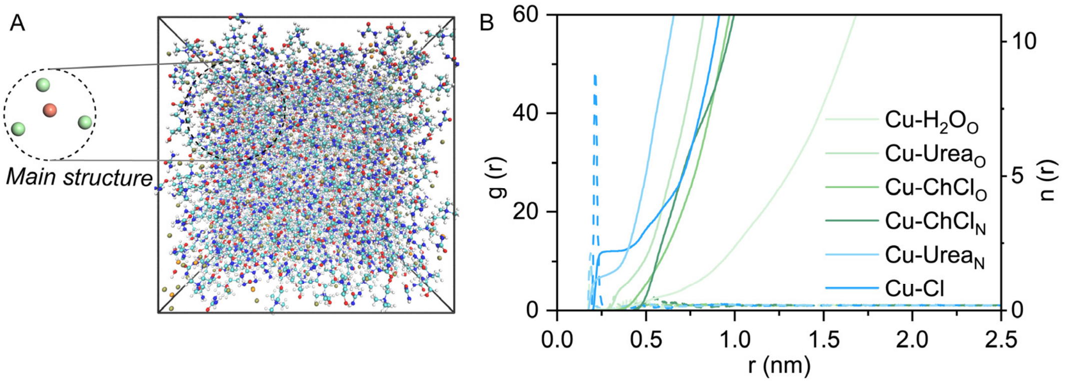

Fig. S5. Thermodynamic analysis and electrochemical validation of the Cu^{2+}/Cu^{+} redox couple in a DES electrolyte. (**A**) The solvation structures of the CuCl/DES. Atom color: Cu, orange; Cl, tan; N, blue; C, gray; H, white; O, red. (**B**) The resulting RDF g(r) and coordination number N(r) in the CuCl/DES.

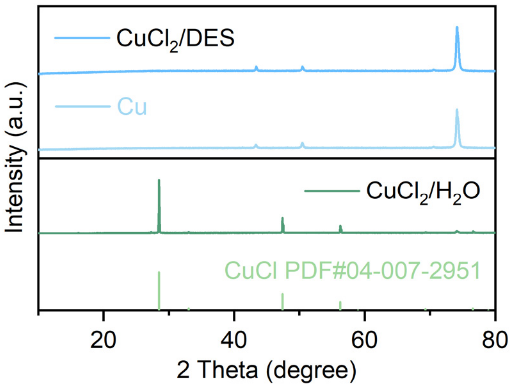

Fig. S6. Thermodynamic analysis and electrochemical validation of the Cu^{2+}/Cu^{+} redox couple in a DES electrolyte. The XRD pattern of the Cu anode cycled in the $CuCl_2$/DES and $CuCl_2/H_2O$ electrolyte.

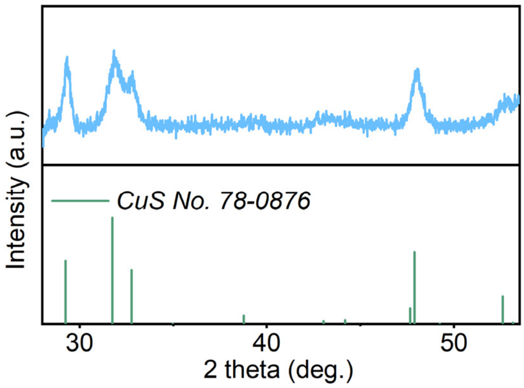

Fig. S7. Working mechanism of the gas-liquid-solid tri-phase redox cascade cell. The XRD pattern of CuS during the discharge process.

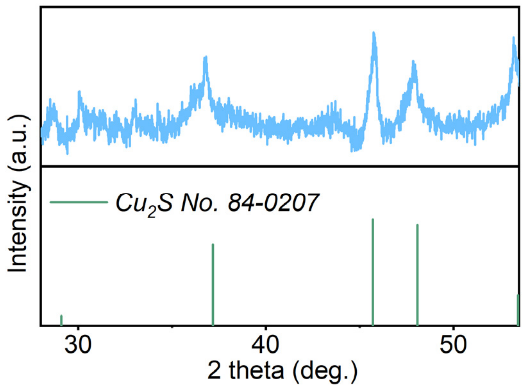

Fig. S8. Working mechanism of the gas-liquid-solid tri-phase redox cascade cell. The XRD pattern of Cu_2S during the discharge process.

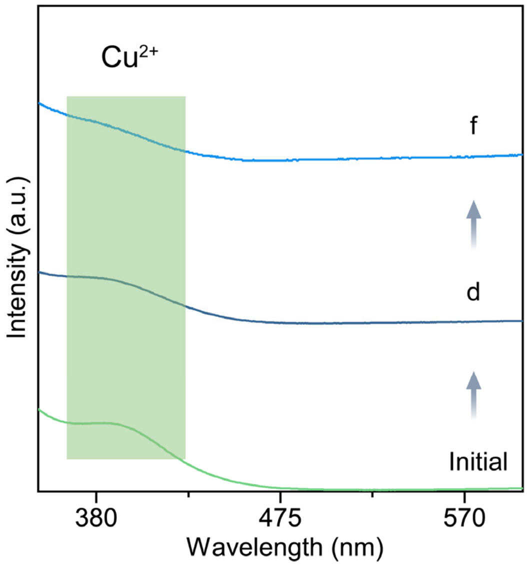

Fig. S9. Working mechanism of the gas-liquid-solid tri-phase redox cascade cell. UV-vis spectra of copper species at initial (point a), discharged to 0.45 V (point d), and fully discharged (point f) states in the cascade cell.

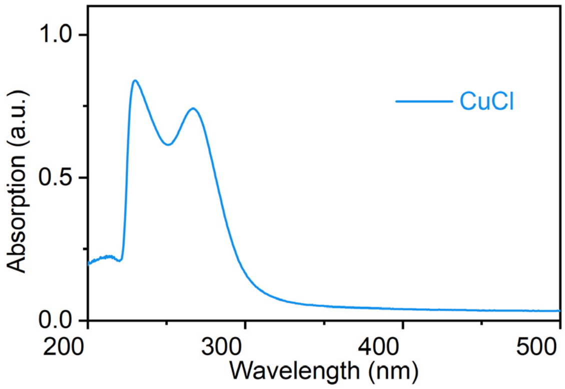

Fig. S10. Working mechanism of the gas-liquid-solid tri-phase redox cascade cell. The UV-Vis spectra of CuCl/DES electrolyte.

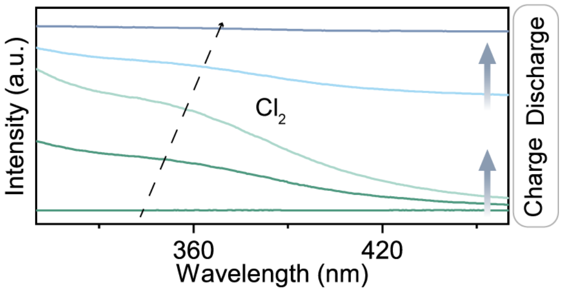

Fig. S11. Working mechanism of the gas-liquid-solid tri-phase redox cascade cell. Reversible Cl_2 transition characterized by UV-Vis spectroscopy.

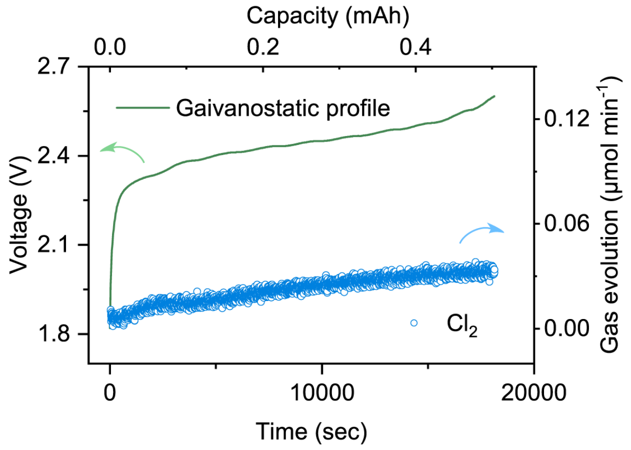

Fig. S12. Working mechanism of the gas-liquid-solid tri-phase redox cascade cell. The gas evolution during the charging process.

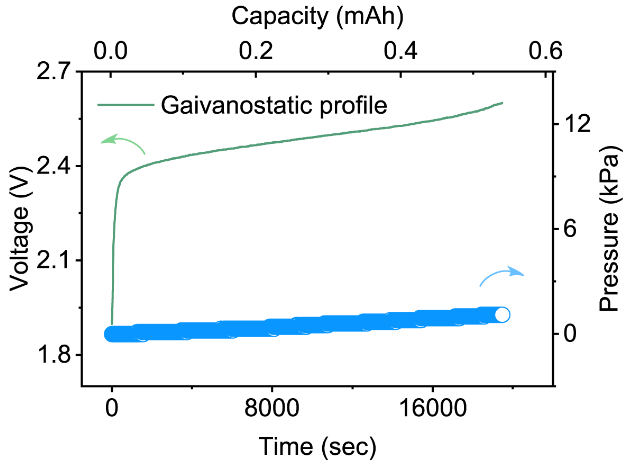

Fig. S13. Working mechanism of the gas-liquid-solid tri-phase redox cascade cell. Evolution of internal cell pressure during the charging process.

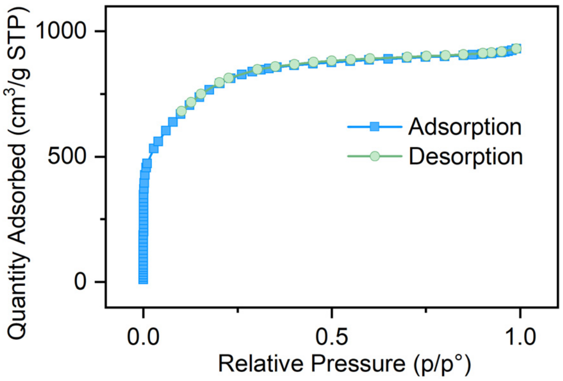

Fig. S14. Working mechanism of the gas-liquid-solid tri-phase redox cascade cell. Adsorption-desorption isotherms of the porous carbon host.

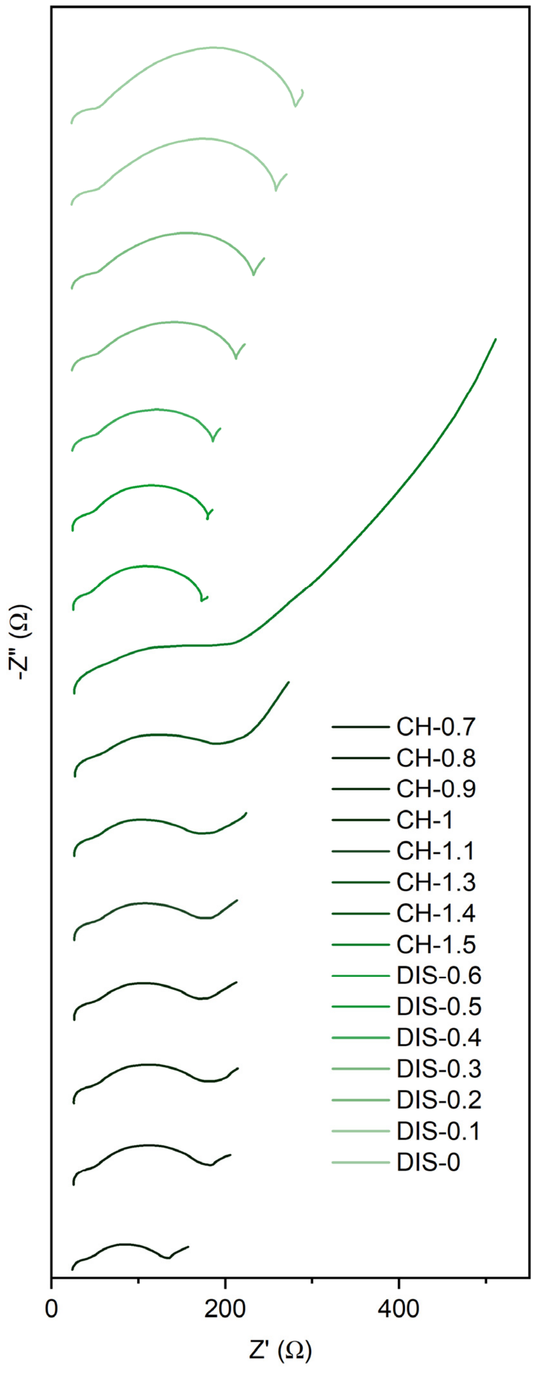

Fig. S15. The electrochemical behaviors of the cascade cell. In-situ EIS of the Cu-C cell.

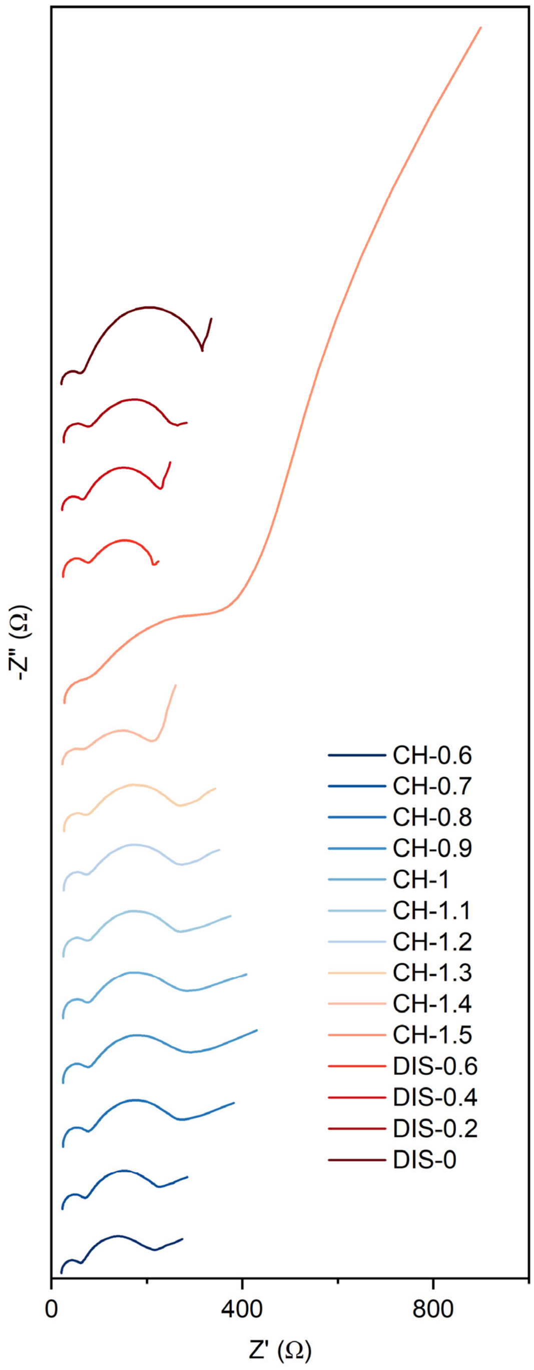

Fig. S16. The electrochemical behaviors of the cascade cell. In-situ EIS of the cascade cell.

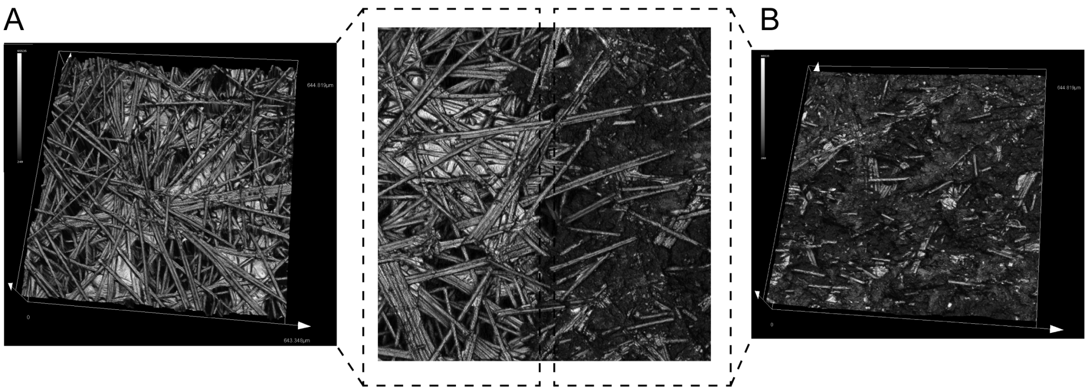

Fig. S17. The electrochemical behaviors of the cascade cell. (**A**) The confocal laser scanning microscope image of the exposed cathode surface. (**B**) The confocal laser scanning microscope image of the cathode surface with S@AC coating.

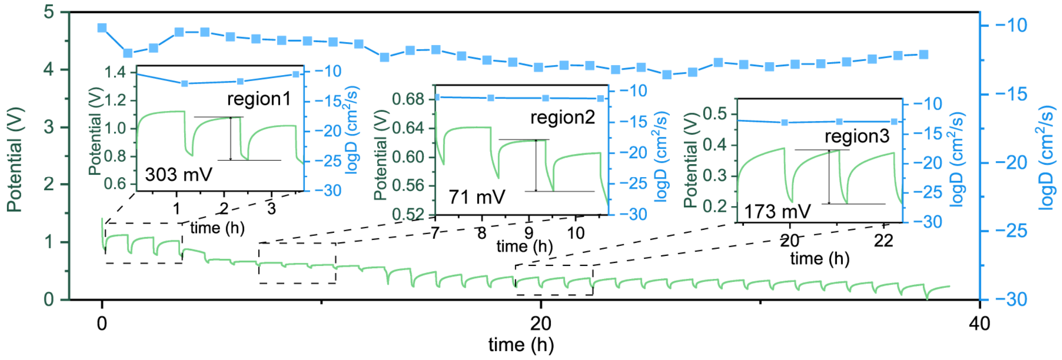

Fig. S18. The electrochemical behaviors of the cascade cell. The GITT of the cascade cell at 1 A g^{-1} (based on sulfur).

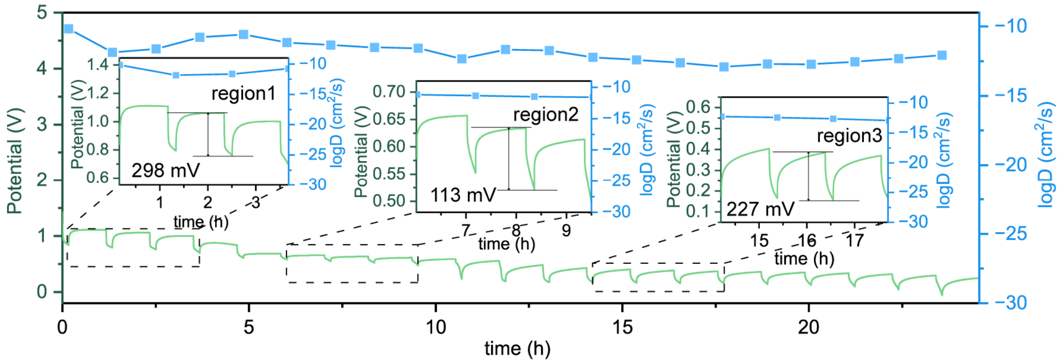

Fig. S19. The electrochemical behaviors of the cascade cell. The GITT of the cascade cell at 2 A g^{-1} (based on sulfur).

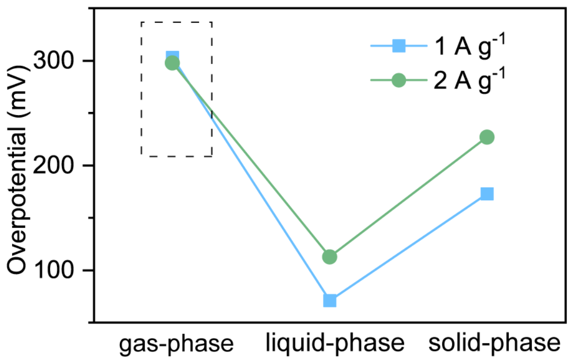

Fig. S20. The electrochemical behaviors of the cascade cell. Comparison of overpotentials across various reaction stages between two applied currents.

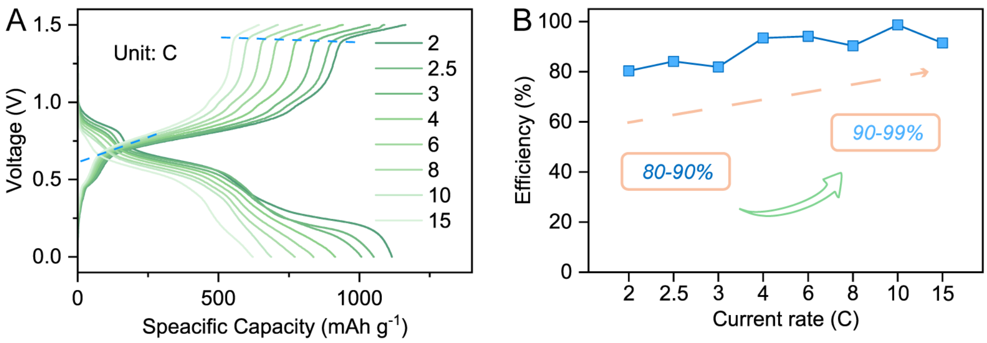

Fig. S21. Electrochemical performance of the cascade cell. (**A**) GCD profiles of the cascade cell at various current rates. (**B**) Coulombic efficiency for the gas-phase redox reaction ($Cl_2 \leftrightarrow Cl^-$) at different current rates.

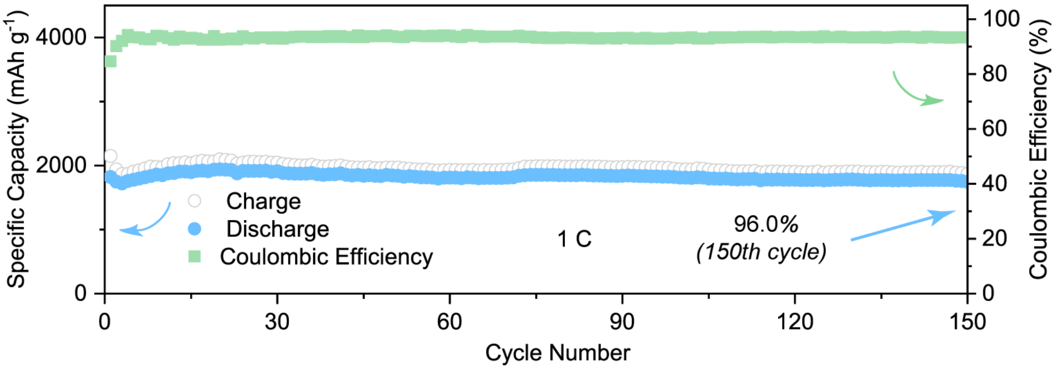

Fig. S22. Electrochemical performance of the cascade cell. The long-term cycling performance of the Cu-S cascade cell at 1 C.

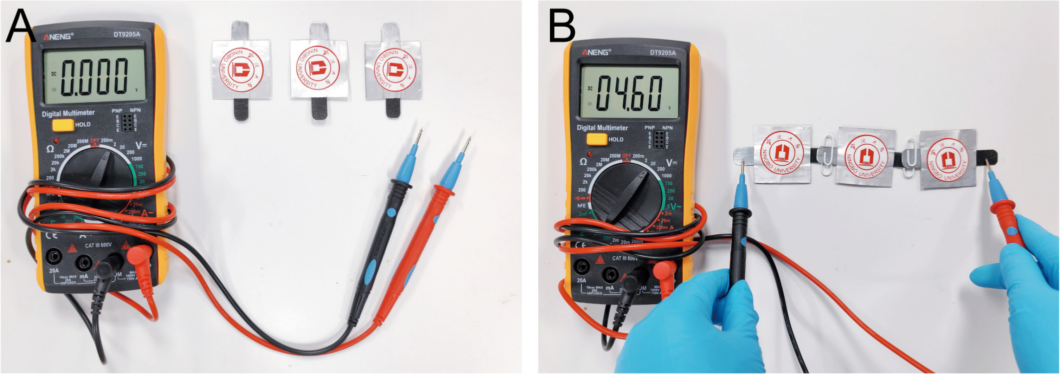

Fig. S23. Practical application of the cascade battery. The open-circuit voltage of the cascade battery.

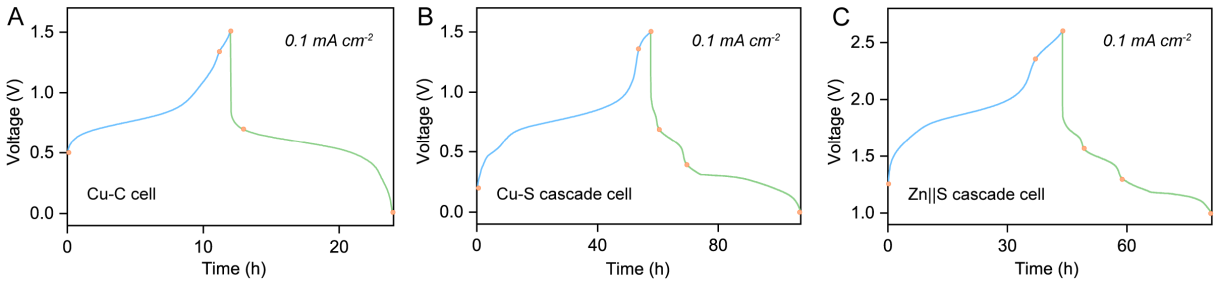

Fig. S24. Practical application of the cascade battery. Charge/discharge voltage profiles vs. time for the (**A**) Cu-C cell, (**B**) Cu-S cascade cell and (**C**) Zn||S cascade cell.

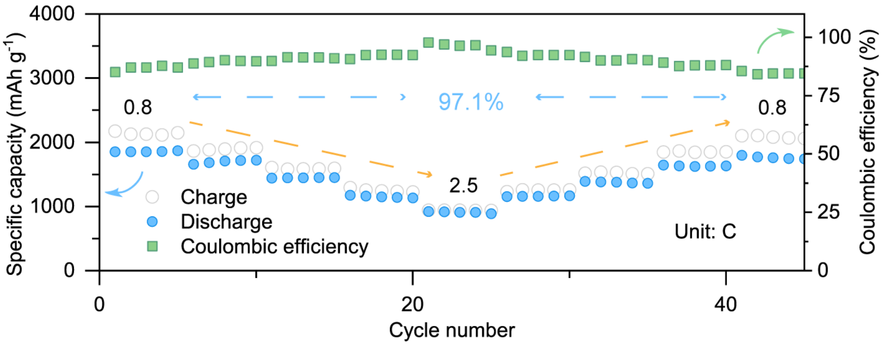

Fig. S25. Practical application of the cascade battery. Rate performance of the pouch cell.

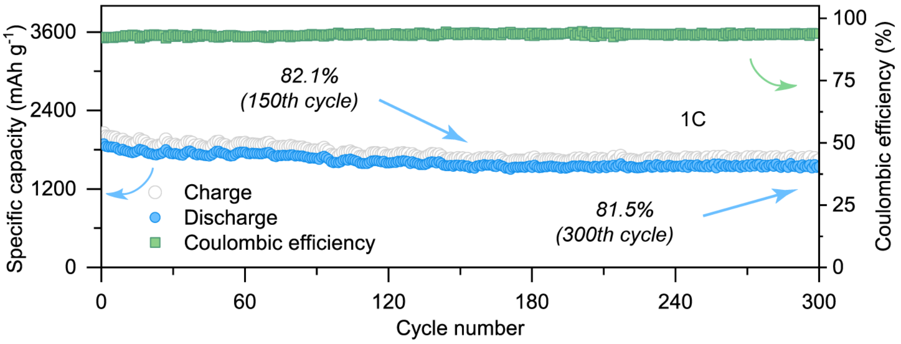

Fig. S26. Practical application of the cascade battery. The long-term cycling performance of the pouch cell at 1 C.

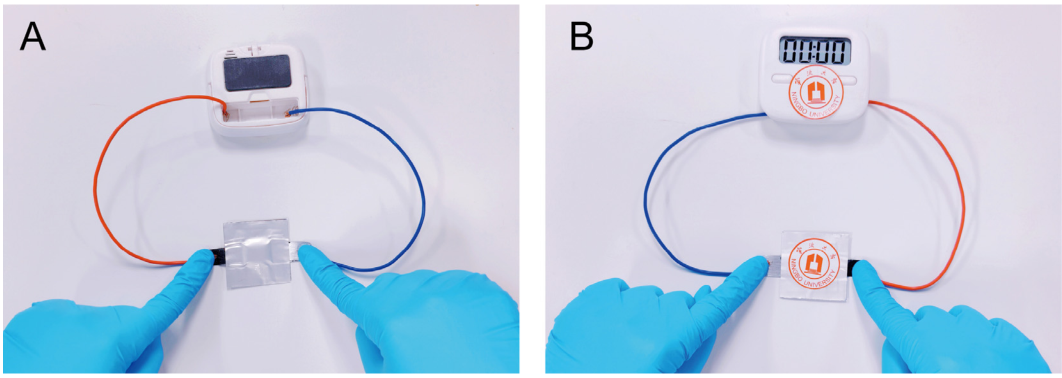

Fig. S27. Practical application of the cascade battery. The pouch cell powers a timer.

Table S1. Summary of electrochemical cell configurations used in this study.

System	Cathode	Anode	Type	Purpose
Cu-S cascade cell	S@AC	Cu	Full cell	Fundamental study of S cathode in cascade system
Zn‖S cascade cell	S@AC	Zn	Full cell	Practical high voltage configuration
Cu-C cell	AC	Cu	Full cell	Investigate the gas-phase and liquid-phase redox reactions
